# Effort Gains in Occupational Teams – The Effects of Social Competition and Social Indispensability

**DOI:** 10.3389/fpsyg.2018.00769

**Published:** 2018-05-22

**Authors:** Guido Hertel, Christoph Nohe, Katrin Wessolowski, Oliver Meltz, Justina C. Pape, Jonas Fink, Joachim Hüffmeier

**Affiliations:** ^1^Organizational and Business Psychology, Department of Psychology, University of Münster, Münster, Germany; ^2^Institut für Psychologie, Technical University of Dortmund, Dortmund, Germany

**Keywords:** social competition, social indispensability, motivation gains in teams, effort gains in teams, event reconstruction method, mood, task meaningfulness, strain

## Abstract

Laboratory research has demonstrated social competition and social indispensability as potential triggers of effort gains in teams as compared to working alone. However, it is unclear whether such effects are also relevant for existing occupational teams, collaborating for longer time intervals and achieving meaningful outcomes. We assumed that social indispensability effects are prevalent and stable in occupational teams, whereas social competition effects should mainly be effective in the beginning of teamwork and fade out over time. Hypotheses were confirmed in two studies using within-subjects designs with employees recruited via an online panel (Study 1, *N* = 137) and in software development companies (Study 2, *N* = 70). By means of the Event Reconstruction Method, participants re-experienced specific events from past working days (three events working alone, three teamwork events), and rated their effort separately for these events. In both studies, multilevel analyses revealed significant effort gains in teams when event-specific social indispensability was high. These effects were mediated by positive mood and perceived task meaningfulness, and additionally qualified by employees’ preference for teamwork. In contrast, motivating effects due to event-specific social competition were only observed for teams with short as compared to long team tenure in Study 2.

## Introduction

Teamwork is an important building block of today’s work organizations (Kozlowski and Ilgen, 2006; Mathieu et al., 2014), with potentially significant consequences for employees’ effort expenditure at work. Indeed, laboratory research has not only demonstrated demotivating effects of teamwork (e.g., Karau and Williams, 1993) but also motivating effects of teams up and beyond the level of working alone (e.g., Williams and Karau, 1991; Weber and Hertel, 2007). However, it is still not clear whether these effects generalize to occupational settings with more meaningful tasks and incentives, and when teams collaborate for longer periods of time (e.g., Erez and Somech, 1996). For instance, motivating features relevant at the beginning of teamwork might lose their potential or even lead to reversed effects over time. In particular, unambiguous documentation of effort *gains*^1^ in teams as compared to working alone is still very rare for existing occupational teams. Moreover, the mediating processes of such effort gains have not been investigated so far.

The present research provides evidence for effort gains in existing occupational teams. In two field studies, we chose a within-subject design that enables comparisons of teamwork and individual work within the same persons, reducing error variance and enabling a conservative test of true effort *gains* in teams. Moreover, a within-subjects design allows for comparing different sources of effort gains in teams within the same persons. Considering event-specific experiences of workers in various branches, we compared social competition and social indispensability effects as currently perhaps best established sources of team effort gains in laboratory research (e.g., Weber and Hertel, 2007; Larson, 2009), and explored affective and cognitive contingencies of these sources as potential mediating mechanisms. Finally, we considered team tenure and person-related dispositions as potential moderating factors.

Thus, this research contributes to the literature in four different ways: first, we conducted one of the first comparisons between teamwork and individual work *in occupational field settings*, assessing if, when, and why teamwork is more motivating for individuals than working alone. In doing so, we built on laboratory research that revealed social competition and social indispensability as potential sources of team effort gains. However, generalizing effects from short-term laboratory research to enduring occupational teamwork is not trivial. For instance, some motivating features might work only in the very beginning of teamwork and fade out over time. Moreover, collaborating with well-known team partners on career-relevant goals and outcomes is quite different from collaborating with unfamiliar (or even unknown) partners on rather short and unimportant tasks in the laboratory (e.g., Aiello and Douthitt, 2001). Second, we examined and compared *multiple sources of effort gains* in occupational teams using a *within-subjects design*, providing a more differentiated analysis of motivating features in teams. For instance, individuals might increase their effort only temporarily when they perceive their contribution to be highly relevant, but might perform at the level of individual work (or even below) otherwise. Such effects are difficult to disentangle in between-subject designs. Third, we measured contingent affective (mood) and cognitive processes (perceived task meaningfulness) in order to explore *mediating mechanisms* of effort gains in teams. Notably, these mediating mechanisms might also have implications for the prevalence of social competition and social indispensability in occupational teams. Finally, we examined *team tenure and individuals’ dispositions as potential moderators* that also speak to the assumed process dynamics. Together, this research contributes to a more differentiated understanding and theoretical conceptualization of microdynamics in teams as an important element of team processes (see Humphrey and Aime, 2014; Kozlowski, 2015, for recent calls). Although motivation is only one out of multiple process variables in occupational teams, individuals’ effort can be seen as a core process that determine a wide variety of other outcomes, such as performance or innovation in teams.

### Motivating Effects of Teamwork

Historically, team effects on individuals’ effort in performance settings have been among the first topics of empirical research in psychology (e.g., Triplett, 1898; Ringelmann, 1913). Although some of these early studies already observed motivating effects of teamwork as compared to working alone (e.g., Moede, 1914; Köhler, 1926), later research predominantly focused on *de-*motivating effects of teamwork, suggesting that perceived dispensability, lack of identifiability, or feelings of being exploited can significantly reduce individuals’ effort in teams (e.g., Karau and Williams, 1993; Shepperd, 1993). Most of these studies have been conducted in laboratory settings with student participants, which limits the generalizability of the findings due to low task meaningfulness, low incentives for the participants, and lacking past or future of the examined teams (e.g., Erez and Somech, 1996). Studies with enduring occupational teams in field settings are still pending that particularly examine effort *gains* in teams as compared to individual work. Existing studies in occupational settings rather focused on the optimization of teamwork, comparing highly successful teams with less successful teams (e.g., Hu and Liden, 2015, as a recent example). While this is quite important for our understanding of teams, the neglected contrast of teamwork with non-teamwork in occupational settings impedes the assessment if, when, and why teamwork can truly exert additional motivation.

According to laboratory research with high control of task and context conditions, two well established sources of effort gains in teams are social competition with other team members (e.g., Stroebe et al., 1996), and social indispensability for the team outcomes (e.g., Hertel et al., 2000; see Weber and Hertel, 2007, for a meta-analytic review). We describe the psychological dynamics of these two mechanisms next, including potential contingencies with individuals’ cognitions and affective states.

### Social Competition as Trigger of Effort Gains in Occupational Teams

The first considered source of effort gains in teams is based on Festinger’s seminal work, conceptualizing social comparison as motivating but also sense-making process in social interactions (e.g., Festinger, 1954). This basic mechanism can be found in various theoretical approaches of social motivation, such as upward comparison (e.g., Major et al., 1991), performance matching (e.g., Jackson and Harkins, 1985), or goal comparison (Stroebe et al., 1996; see also Bandura and Cervone, 1983), and is also the core concept of social competition in teams (e.g., Seta, 1982). Social competition is often based on agentic values (“getting ahead”), striving for mastery and social dominance as compared to communal values (“getting along”) related to cooperation and the fulfillment of social bonds (e.g., Trapnell and Paulhus, 2012). In teams, competition with other team members can provide performance standards for an individual and affect consecutive effort expenditure (e.g., Wittchen et al., 2011; Lount and Wilk, 2014). For instance, when individual members perceive other team members to be more successful in a valued task, they should increase their personal performance goals in order to match or even exceed the performance of the other team members (e.g., Stroebe et al., 1996). Indeed, laboratory studies have documented that social competition can lead to significant effort gains in teams as compared to working alone (e.g., Seta, 1982; Stroebe et al., 1996; for meta-analytic data, see Weber and Hertel, 2007). The basic mechanism should not be limited to teamwork in laboratory settings, but might also lead to effort gains in enduring occupational teams with meaningful tasks and outcomes. Thus, we assume:

H1: Social competition in occupational teams predicts higher effort of individual workers as compared to working alone.

A recent field study by Lount and Wilk (2014) provided initial evidence that social competition might indeed trigger significant effort gains in enduring occupational teams. The authors compared employees of a call center when working alone and when working on a project team using a within-subjects design. Consistent with a social comparison or competition approach, teamwork led to higher performance as compared to working alone when social competition was made very salient during a 6-week period of performance posting (i.e., individual workers’ performance data were posted in the office). However, workers’ team performance dropped even *below* the level of working alone when performance posting was ceased after 6 weeks. Moreover, these effort losses were similar to observed effort losses in teams before the explicit performance posting intervention, suggesting that the observed effort losses were not merely a reaction to the removal of performance postings but rather the regular status quo. Thus, although providing initial evidence for social competition as a source of effort gains in enduring occupational teams, the reported effect seems to be rather fragile and contingent on additional context conditions (i.e., explicit posting of performance data). Moreover, the underlying psychological processes remain unclear because the authors analyzed performance data only. More in-depth research is desirable that also considers process variables (e.g., affect, cognitions) and potential moderators.

Finally, Lount and Wilk (2014) focused only on social competition as an agentic and rather individualistic motivation (maximizing individual gains). In order to achieve a more complete picture of the motivating potential of occupational teamwork, it might be promising to consider also sources of effort gains that are related to more collectivistic motivation (maximizing team gains) based on communal values (Trapnell and Paulhus, 2012) and other-oriented motives (e.g., De Dreu, 2006). Next, we describe a potential source of effort gains in teams that is connected to mutual interdependence as a central feature of teamwork.

### Social Indispensability as Trigger of Effort Gains in Occupational Teams

Social indispensability effects in teams are assumed to occur because the perception of being important for the team outcome increases the perceived impact and meaningfulness of an individual’s contribution (e.g., Karau and Williams, 1993; Hertel et al., 2000). This effect is reflected and additionally fueled by social norms prescribing to support the team one belongs to, and not to let down the other team members (loyalty or generic ingroup norms; e.g., Tajfel, 1970; Hertel and Kerr, 2001). Motivating effects of being indispensable, and thus responsible for others, are also considered in concepts of intrinsic motivation (e.g., “meaningfulness for others”; cf. Hackman and Oldham, 1976), prosocial motivation (e.g., Grant and Berg, 2011), or the centrality of contributions in social dilemmas (e.g., Au et al., 1998). Consistent with established instrumentality × value models of motivation in teams (e.g., Karau and Williams, 1993), we assumed that individuals are quite sensitive to whether or not their individual effort is important for the team outcome. When individuals perceive their contribution to a team outcome as dispensable, they should reduce their efforts (“free riding”; e.g., Kerr, 1983; Kerr and Bruun, 1983). However, when individuals perceive their contribution to a team outcome as indispensable, they should increase their efforts even beyond the level of working alone (Hertel et al., 2000).

As with social competition, laboratory research has provided evidence that social indispensability (or responsibility) for a team can trigger significant effort gains as compared to working alone. For instance, being the weaker partner in a conjunctive performance dyad (in which the weaker person’s performance determines the dyad’s overall outcome; Steiner, 1972) can increase individuals’ effort by 30% and more as compared to working alone (e.g., Hertel et al., 2000, 2008; Kerr et al., 2007). Notably, these social indispensability effects occurred even when social competition effects were controlled for (e.g., Weber and Hertel, 2007). However, team cooperation in these studies did not exceed 1 h in time, cooperation partners were usually foreigners (and sometimes even simulated by a computer algorithm), and the consequences of successful cooperation included only relatively small monetary incentives (Kerr and Hertel, 2011). A more recent laboratory study has provided evidence that social indispensability effects can be found across five trials of an aerobic exercise task (up and beyond mere social competition effects; see Irwin et al., 2012). However, unambiguous evidence for social indispensability as trigger of effort gains in enduring occupational teams and everyday working contexts is still pending.

Based on the rationale described, we assumed that being indispensable for the results of an occupational team should lead to similar or even higher effort gains in teams as in laboratory research, given that meaningful outcomes, longer periods of cooperation, and acquaintance with fellow team members all increase the importance of the teamwork results. Therefore, we expected:

H2: Social indispensability for the team outcome in occupational teams predicts higher effort of individual workers as compared to working alone.

Initial evidence for social indispensability effects in field settings with existing teams stems from analyses of archival data of swimming competitions at major sport events, documenting that athletes swam faster in relays as compared to individual competitions when their contribution to the relay was highly indispensable (i.e., starting at the last relay position; e.g., Hüffmeier and Hertel, 2011b). However, generalizability of these findings to regular work settings might be limited due to the extreme preselection of individuals (i.e., only the most successful competitive swimmers of the world) and the specific type of teamwork (sequential collaboration in action teams; e.g., Sundstrom, 1999). Moreover, the focus on maximum instead of typical performance might even underestimate motivational influences on performance (e.g., Klehe and Anderson, 2007). Finally, the analyses of archival data included performance measures only, and provide no indicators of mediating psychological processes.

Aside from analyses of swimming relays, existing studies with occupational teams have considered social indispensability only when comparing teams with other teams (e.g., Hertel et al., 2003b, 2004), but not when comparing teamwork with working alone. Moreover, within-person variance of effort as a function of social indispensability has not been examined in work settings at all. For instance, team members might increase their effort temporarily when they perceive their contribution to be particularly important for the team, but might perform at the level of individual work (or even below) otherwise. Such motivating events are difficult to discover in between-subject designs when individual performance is aggregated across time. A within-subject design enables more specific analyses of motivating and demotivating aspects of different job events for the same individual worker.

### Potential Mediating Mechanisms of Effort Gains in Teams

In addition to comparing social competition and social indispensability effects in occupational teams, we also examined affective (i.e., mood state) and cognitive (i.e., perceived meaningfulness of the current task) contingencies as potential mediating mechanisms of these effects. Affective states have already been discussed as potential mediators of social indispensability effects (e.g., Weber and Hertel, 2007; Hertel et al., 2008). Indeed, both the pioneering work of Köhler (1926) as well as more recent laboratory studies (e.g., Hertel et al., 2000, 2003a) showed that social indispensability was accompanied with positive mood states. Theoretically, fulfilling responsibilities for a team should be associated with various positive consequences, such as being acknowledged and accepted as a group member. Indeed, in a recent study, Fung et al. (2016) found that endorsing communal values (but not agentic values) was positively associated with subjective well-being. In addition, working together with others and anticipating the success of the whole team can be associated with feelings of enjoyment and pride. Anticipating and experiencing such positive consequences might elevate persons’ mood state, which in turn might increase persons’ tolerance for unpleasant experiences (e.g., fatigue or pain) and enable higher effort expenditure (Stanne et al., 1999; LePine and Van Dyne, 2001). Thus, being indispensable for a team might elevate (or at least maintain) individuals’ mood, and increase (or maintain) the willingness to exert effort and/or the subjective threshold to quit trying (e.g., Martin et al., 1993).

Positive mood (or the anticipation thereof) might be also considered as a mediator of social competition effects on individual effort in teams. However, this process is probably more fragile and qualified by the relative capability of an individual. Whereas outperforming others might elevate ones mood, being outperformed by others should rather lead to negative feelings, particularly when these unfavorable comparisons occur repeatedly with the same persons (Kemmelmeyer and Oyserman, 2001; Brown et al., 2007). Thus, social competition within the team can also lead to frustration for a considerable part of the members (e.g., Stanne et al., 1999; Mendes et al., 2001). Moreover, social competition might lose its affective value even for stronger team members when working repeatedly with the same persons (e.g., Lount et al., 2008). As a consequence, we expected that social competition is overall only weakly (if at all) correlated with positive mood in occupational teams. Together, we postulated:

H3a: Effects of social competition on effort gains in occupational teams are partially mediated by positive mood.
H3b: Effects of social indispensability on effort gains in occupational teams are partially mediated by positive mood.
H3c: The relationship between social indispensability and positive mood is stronger than the relationship between social competition and positive mood in occupational teams.

In addition to affective states, we considered the perceived meaningfulness of the current working task as a potential cognitive mediator of effort gains in teams. In general, the more meaningful a task is perceived, the more effort an individual should invest (e.g., Hackman and Oldham, 1980). Social indispensability should increase the perceived meaningfulness of a task because supporting the team is usually followed by positive consequences, such as acknowledgment, thankfulness, and potential reciprocity from the other team members. Failing to support the team when a contribution is highly needed (“letting the team down”), in contrast, is followed by negative social sanctions. In addition to individualistic concerns (e.g., avoiding negative sanctions from others), indispensability perceptions should particularly increase the perceived meaningfulness of a task due to communal values and collectivistic concerns (maximizing joint gains; e.g., Weber and Hertel, 2007; Hertel et al., 2008). Such concerns for others are often neglected in applied psychology, but might provide important supplements in the explanation of motivation at work (e.g., De Dreu, 2006; Grant and Berg, 2011; Hu and Liden, 2015). Notably, effort gains due to social indispensability have been shown even when team members were not identifiable and social sanctions not possible (e.g., Hertel et al., 2003a, 2008).

Social competition might also increase the perceived meaningfulness of a task because additional outcomes can be achieved, such as recognition, social status, or monetary compensation (Festinger, 1950). This is particularly the case in new work settings (e.g., a new project team) when lacking performance standards are replaced by subjective comparisons among the workers involved. However, this positive connection between social competition and perceived meaningfulness might again vary as a function of team members’ relative capabilities. For instance, team members might devalue tasks when they believe to have only few capabilities. In addition, social competition might lose its informative value for more capable team members when working repeatedly with the same persons (e.g., Lount et al., 2008). In contrast, social indispensability and related responsibility for others should yield personal meaningfulness to contributions regardless of time and the relative capabilities of team members. Thus, we expected that social indispensability in occupational teams is more strongly correlated with perceived task meaningfulness than social competition:

H4a: Effects of social competition on effort gains in occupation teams are partially mediated by perceived task meaningfulness.
H4b: Effects of perceived indispensability on effort gains in occupational teams are partially mediated by perceived task meaningfulness.
H4c: Perceived indispensability in occupational teams is more strongly correlated with perceived task meaningfulness than social competition.

The distinction between social competition and social indispensability as different sources of effort gains in occupational teams has not only implications for the subjective experience of team members, but also for the stability and prevalence of effort gains in teams over time. In general, we assumed that social indispensability effects are more stable, and thus more prevalent in occupational teams because they are – on average – more strongly correlated with positive mood and perceived task meaningfulness for the acting individual (see H3–4). In addition to basic reinforcement effects, these positive experiences might also provide additional psychological resources (e.g., coping resources) that additionally foster the stability of effort gains in teams. Moreover, the assumed contingencies of positive mood and perceived task meaningfulness with social competition in teams might be restricted to team members with relatively high capabilities, whereas no such restrictions are assumed for social indispensability effects. Indeed, fulfilling normative demands to act responsibly and reliably for a team is rather independent of the relative capabilities of team members. Finally, while the informative value of social competition might fade out over time, meeting individual responsibilities in a team should remain important over time. Based on these considerations, we expected:

H5: Social indispensability is a stronger source of effort gains in occupational teams than social competition.

### Potential Moderators of Effort Gains in Teams

In addition to potential mediating processes of effort gains in teams, we also examined both context- (i.e., team-) and person-related moderators of these processes. As outlined above, *team tenure* should moderate effects of social competition on effort gains in teams because social comparison is particularly relevant in new social settings (e.g., Festinger, 1954). However, both informative and affective values of social competition should wear off over time in repeated interactions with the same partners (e.g., Lount et al., 2008). Therefore, we expected:

H6: Team tenure moderates effort gains in occupational teams such that effort gains due to social competition are stronger when team tenure is short as compared to long.

No such moderation was expected for social indispensability because both affective and cognitive mediation processes are assumed to hold for both short and long team tenure. Among the person-oriented moderators, we considered event-specific self-efficacy as potential moderator of the relation between social competition and social indispensability with effort gains in teams. As outlined above, social competition might be more motivating for team members with high as compared to low capabilities because the former experience more positive affect in social competitions and are less likely to devalue social competition in teams. In a similar way, high task capabilities might be a precondition for perceived indispensability in teams to be highly motivating. However, event-specific self-efficacy might also moderate effort gains in teams in a curvilinear (inverted U) pattern, for instance, because social competitions are particularly meaningful when self-efficacy is medium rather than high or low (e.g., Festinger, 1954). Thus, we refrained from postulating hypotheses here and examined event-specific self-efficacy in an exploratory way.

Finally, we also considered person-oriented moderators that crystallize the assumed main processes in order to further examine the underlying rationale described above. We expected social competition effects to be stronger for persons with a competitive orientation, placing high values on social comparisons with others (Smither and Houston, 1992). In contrast, we expected social indispensability effects to be stronger for persons who prefer teamwork instead of working alone (Karau and Elsaid, 2009). These persons should be more likely to endorse communal values and follow collectivistic orientations (e.g., maximizing team outcomes), and should thus be more susceptible for social responsibility norms not to let the group down. Thus:

H7: Individuals’ preferences for social competition moderate effort gains in occupational teams such that effort gains due to social competition are stronger for individuals with a high as compared to a low preference for social competition.
H8: Individuals’ preferences for teamwork moderate effort gains in occupational teams such that effort gains due to perceived indispensability are stronger for individuals with a high as compared to a low preference for teamwork.

Please note that the latter two hypotheses are not trivial. In fact, it might also be that situational triggers of social competition in teams are particularly motivating for individuals with a *low* preference for social competition because individuals with a high preference for social competition compare themselves regardless of situational triggers. Similarly, it might be that situational triggers of social indispensability in teams are particularly motivating for individuals with a *low* preference for teamwork because individuals with a high preference for teamwork might feel responsible for their fellow team members even without specific situational cues.

The hypotheses were tested in two studies with employees working both alone and on a team. In both studies, participants were instructed to reconstruct and re-experience six specific job events from the last few days (three events when working on a team, and three events when working alone) following the Event Reconstruction Method as a non-invasive procedure to capture within-person variations of perceptions and experiences at work (Grube et al., 2008; Hertel and Stamov-Rossnagel, 2013). The data allowed to compare participants’ effort during teamwork and when working alone within the same person and contingent on event-specific variations of perceived indispensability and social competition in the team. Finally, dispositional moderators were examined in Study 2.

## Study 1

### Method

#### Participants

Study 1 was conducted as web-based survey with employees contacted via an online panel. In general, online panels consist of persons who have voluntarily registered to participate repeatedly in web-based studies, enabling short field times and high data quality (e.g., Göritz and Luthe, 2012). The participants of this online panel had registered to participate in psychological studies out of personal interest and received no compensation for their participation in the study. Out of 1.073 invited panelists, 151 persons completed the questionnaire and agreed that their data could be used for scientific purposes. Please note that this return rate can be considered as normal for online panels with voluntary participants (e.g., Göritz and Crutzen, 2012) and with a time-consuming questionnaire, and should not be compared with return rates of employee surveys where participants are invited by their managers, answer during payed working time, and expect that the survey results improve their personal working conditions. Teamwork was not mentioned as research theme in the invitation of participants. In the following analyses, we excluded participants who were currently unemployed (*N* = 10), who reported very low rates of teamwork (5% or below of the weekly working hours, *N* = 3), and who did not follow instructions by reporting the same date for all six work events (*N* = 1).^2^ The final sample contained 137 participants (78 women, 59 men; average age of 46.3 years, *SD* = 9.5, age range 24–63 years), with 51.8% holding a university degree and 48.2% having completed several years of professional training. The occupational fields represented in the sample include healthcare (22.6%), service (22.6%), governmental sector (19.7%), media and IT (13.9%), industry (8.8%), and education (4.4%). Participants reported a mean organizational tenure of 12.6 years (*SD* = 10.7), and spend on average 41.8% (*SD* = 25.6) of their working time in teams.

#### Procedure

The study was introduced as exploration of motivation in different work contexts. Explicit definitions of teamwork and working alone were provided to ensure a clear understanding of the two different settings:

Teamwork: When working on a team, you work together with one or more colleagues on a shared task, and have to arrange and coordinate the subtasks among you.
Working alone: When working alone, you work independently of others and are solely responsible for the execution of the task. Therefore, you don’t need to arrange and coordinate your work with other colleagues (translated from German).

Then, participants were instructed to reconstruct and re-experience specific job events from the last working days following the Event Reconstruction Method (ERM; Grube et al., 2008; Hertel and Stamov-Rossnagel, 2013):

Please think about an event of the last working days in which you worked in a team with others [in which you worked individually]. Please take a moment to put yourself in this situation. Recall precisely what you have done (translated from German).

Building on the Day Reconstruction Method (Kahneman et al., 2004), the ERM utilizes specific trigger questions (e.g., “Who was present?”, “Where did the event happen?”) to activate episodic memory traces that provide rich and vivid access to experienced affect and cognitions “*in situ*” without being as invasive as experience sampling methods (e.g., Csikszentmihalyi and LeFevre, 1989). Participants were asked to reconstruct three recent team events and three recent events working alone. When working in more than one team simultaneously, participants were asked to refer to the team in which they worked most of the time. Half of the participants started reconstructing a block of three team events followed by a block of three events working alone, while the other half started with the reversed order of blocks. Participants were randomly assigned to the two order conditions. For each reconstructed event, participants indicated date and time of the day.

#### Measures

Perceived effort expenditure as well as mood and perceived meaningfulness of work were measured separately for each reconstructed job event. The considered sources of effort gains in teams were measured only for reconstructed team events. Perceived indispensability for the team (“How important was your contribution for the team’s success?”) and social competition with other team members (“How much did you want to be better or at least not worse than others in your team”?) were measured with one item each on a 7-point Likert scale from 1 (not at all) to 7 (very). Both items were adopted from Hertel et al. (2003a). Mood was measured with a Kunin scale (Kunin, 1955) displaying seven different smiley faces ranging from very sad to very happy. Perceived task meaningfulness was measured with one item (“As how meaningful did you perceive your momentary task for yourself?”) on a 7-point Likert scale ranging from 1 (not at all) to 7 (very).

For exploratory reasons, we also measured event-based self-efficacy with one item (“How capable have you felt for your task?”), and event-based strain with one item (“How much pressure did you feel?”) from the Stress in General Scale (Stanton et al., 2001). In occupational teams, social competition and social indispensability might not only trigger additional effort but also lead to strain and even burnout in the long run. At the same time, however, buffer mechanisms might prevent such negative effects. For instance, individuals might change reference groups when social competitions become frustrating. And being indispensable for a team can lead to positive affect, recognition, and social support in return, which might help to cope with additional stress. Thus, we refrained from formulating hypotheses for social competition and social indispensability effects on event-based strain but examined these questions in an exploratory fashion for theoretical and practical reasons.

Finally, effort expenditure was measured for each event with one item (“*How do you rate your work motivation on a scale from 0 [not at all motivated] over 100 [motivated on average] to 200 [extremely motivated]?*”). In order to compare the rated effort in the different team events with a working alone baseline, we compared the rated effort in each team event with an aggregated effort baseline developed from the three working alone events. Thus, for each participant we used the averaged effort rating across the three working alone events as comparison baseline for each of the different team events. The main dependent variable of this study, “effort gains in teams,” was calculated by subtracting participants’ average effort ratings in the working alone events from her/his effort ratings in each of the three teamwork events, resulting in three difference scores of effort gains in teams for each participant (please note that negative values of the “effort gains in teams” variable indicate effort *losses* in teams as compared to working alone). In the end of the questionnaire, participants indicated their age, gender, percentage of working hours spend in teamwork, and team tenure with respect to the focused team. Overall, Study 1 contained 43 items. Moreover, 25 additional items were collected in this survey that were part of a different research question.

#### Data Analyses

Since the data were hierarchically structured within job events (Level 1, *N* = [3 teamwork events × 137] = 411 events) nested within employees (Level 2, *N* = 137 employees), we used multilevel modeling and conducted analyses using Mplus 7.3 (Muthén and Muthén, 1998-2012) with robust maximum-likelihood estimation (MLR). To compare models, we used a scaled log-likelihood difference test (Satorra, 2000) as difference testing in the regular way cannot be applied to models using the MLR estimator (Muthén and Muthén, 1998-2012).

### Results

Descriptive statistics and inter-correlations of Study 1 variables are reported in **Table 1**. As can be seen, questionnaire order was correlated with our main dependent variable. Participants who first re-experienced job events when working alone reported overall effort gains in teams (*M* = 8.75, *SD* = 32.59), whereas participants who first re-experienced teamwork events reported overall effort losses in teams (i.e., negative effort gains in teams: *M* = -10.85, *SD* = 31.79), *t*(135) = 3.57, *p* < 0.001, *d* = 0.57. Notably, examining cross-level interactions revealed that questionnaire order did not moderate event-based effects of social competition or social indispensability on effort gains in teams. Nevertheless, we considered questionnaire order as control variable in the following analyses. Intra-class correlations (ICC) indicated a substantial amount of variance at the within-person level for effort gains in teams (57.3%), social competition (38.8%), social indispensability (69.5%), mood (48.0%), perceived task meaningfulness (59.1%), and strain (45.4%), supporting the use of multilevel analyses.

**Table 1:** Means, standard deviations, and correlations between study variables (Study 1; *N* = 137 Employees).

	Mean	*SD*	1	2	3	4	5	6	7	8	9	10	11	12	13
**Person-level variables**
(1) Order	0.48	0.50	–
(2) Gender	0.57	0.50	–0.05	–
(3) Age	46.32	9.50	0.02	0.01	–
(4) Working time in teamwork^a^	41.81	25.58	0.05	–0.15	–0.02	–
(5) Team tenure^b^	7.56	8.07	–0.01	–0.10	0.38***	–0.01	–
**Event-level variables**
(6) Social competition	4.70	1.58	–0.06	–0.03	–0.05	0.01	–0.20*	–	0.23***	0.01	0.18***	0.22***	0.00	0.11*	0.12*
(7) Social indispensability	5.27	1.11	0.09	0.16	0.02	0.03	–0.02	0.18*	–	0.33***	0.62***	0.11*	0.24***	0.45***	0.31***
(8) Mood	4.85	1.06	0.03	0.09	–0.04	0.03	0.09	–0.07	0.31***	–	0.43***	–0.34***	0.33***	0.69***	0.32***
(9) Meaningfulness	4.86	1.33	0.14	0.13	0.01	–0.01	–0.02	0.11	0.66***	0.44***	–	0.17**	0.21***	0.60***	0.30***
(10) Strain	3.25	1.56	–0.09	–0.01	–0.19*	0.10	–0.24**	0.25**	0.09	–0.39***	0.10	–	–0.35***	–0.12*	0.10*
(11) Self-efficacy	5.88	0.92	0.09	0.14	0.09	0.06	0.18*	–0.03	0.30***	0.36***	0.23**	–0.36***	–	0.21***	0.03
(12) Motivation in teamwork	128.24	39.59	–0.04	0.11	0.07	–0.03	0.03	0.05	0.40**	0.71***	0.62***	–0.23**	0.19*	–	0.57***
(13) Effort gains in teams	–0.62	33.69	–0.29***	–0.01	–0.01	0.05	–0.04	0.06	0.16	0.12	0.13	0.10	–0.13	0.34***	–

*Correlations below the diagonal are correlations at the person-level (N = 137). Event-level data was averaged across the 3 teamwork events. Correlations above the diagonal are correlations at the event-level (N = 411). Order (0 = start with individual work, 1 = start with teamwork), gender (0 = male, 1 = female). ^a^Measured in percentage. ^b^Measured in years. ^∗^p < 0.05, ^∗∗^p < 0.01, ^∗∗∗^p < 0.001.*

#### Social Competition, Social Indispensability, and Effort Gains in Teams

We followed Hofmann and Gavin (1998) and centered within-person predictor variables around each persons’ mean value. Hypothesis 1 stated that event-level social competition predicts effort gains in occupational teams, whereas Hypothesis 2 stated that event-level social indispensability predicts effort gains in occupational teams. Moreover, Hypothesis 5 proposed that social indispensability is a stronger source of effort gains in occupational teams than social competition. These proposed effects were tested in two steps. In the first step, we regressed event-specific effort gains in teams simultaneously on event-specific social competition and social indispensability ratings controlling for questionnaire order. In this analysis, social indispensability positively predicted effort gains in teams (*b* = 12.89, *SE* = 2.01, *p* < 0.001), in line with Hypothesis 2, whereas the relation between social competition and effort gains in teams was not significant (*b* = 2.57, *SE* = 1.74, *ns*), lending no support to Hypothesis 1. Notably, these effects were not qualified by participants’ gender as indicated by non-significant cross-level interaction terms (*b*’s = 1.76 and -3.09, *ns*).

Moreover, team tenure did not qualify the relation between social competition and effort gains in teams (*b* = 0.17), lending no support for Hypothesis 6. Team tenure did also not moderate the relation between social indispensability and effort gains in teams, *b* = -0.10, *ns*. Finally, event-based self-efficacy did not moderate the relation between social competition or social indispensability and effort gains in teams, *b*’s = -1.89 and 0.17 for the linear moderation, and *b*’s = -0.95 and -0.87 for the curvilinear moderation, respectively.

As a more direct comparison of social competition and social indispensability as event-based predictors of effort gains in teams, we constrained the relationships of the two predictors with effort gains in teams to be equal, and compared this constrained model with the unconstrained model. The unconstrained model [-2 × log-likelihood (6) = 4077.55, scaling correction factor = 1.47] fitted the data better than the constrained model [-2 × log-likelihood (5) = 4095.71, scaling correction factor = 1.50], as indicated by a significant log-likelihood difference test [scaled Δ-2 × log-likelihood (1) = 13.73, *p* < 0.001]. Thus, the two relationships differed significantly in magnitude, with social indispensability being the stronger predictor of effort gains in teams as compared to social competition, supporting Hypothesis 5.

In a second step, we examined whether high degrees of event-based social competition and/or social indispensability resulted in positive effort gains in teams, i.e., higher effort in team events as compared to working alone. While the overall difference between effort in teams and effort when working alone was close to zero (*M* = -0.62), the variance of this difference score was considerable (*SD* = 33.69; see **Table 1**) indicating the presence of both effort gains and losses in teams. Therefore, we analyzed effort gains in teams separately for team events with high and with low perceived social competition, and for team events with high and with low perceived indispensability (both separations being based on median-splits). Effort gains in team events with high perceived indispensability were clearly positive (*M* = 11.93, *SE* = 3.23) and significantly higher than zero, *t*(100) = 3.70, *p* < 0.01 (with zero indicating equal effort in teams and in working alone events). However, effort gains in team events with low perceived indispensability were negative (*M* = -14.91, *SE* = 3.86) and significantly lower than zero, *t*(104) = -3.87, *p* < 0.01, indicating factual effort *losses* in those team events as compared to working alone. Thus, in addition to the positive interrelation between social indispensability and effort gains in teams, effort in teams was significantly higher (or lower) than the working alone baseline when social indispensability was high (or low) in the occupational teams. For team events with high levels of social competition, no significant effort gains were observed (*M* = 5.14, *SE* = 3.47), *t*(105) = -1.48, *ns*. However, for team events with low levels of social competition, effort was significantly lower than the working alone baseline, indicating factual effort losses in teams (*M* = -8.22, *SE* = 3.89), *t*(81) = -2.12, *p* < 0.05.

#### Testing Mediation Hypotheses

In order to explore the psychological dynamics triggered by social competition and social indispensability effects in teams, we considered event-based affective and cognitive states as potential mediating mechanisms of social competition and social indispensability effects on effort gains in teams (Hypotheses 3–4). Of course, longitudinal data would be desirable to test such mediation processes. However, the current data allowed at least an examination of contingencies between observed effort gains in teams and the assumed mediator variables as initial indicators of mediation.

We had assumed that social competition effects on effort gains in teams are partly mediated by mood (Hypothesis 3a) and perceived task meaningfulness (Hypothesis 4a). However, social competition did not predict effort gains in teams in Study 1 when social indispensability was accounted for. Therefore, we refrained from further examining potential mediation processes for social competition. Instead, we included perceived social competition as control variable when examining mediation processes predicted by social indispensability effects (Hypotheses 3b and 4b).

**FIGURE 1:**
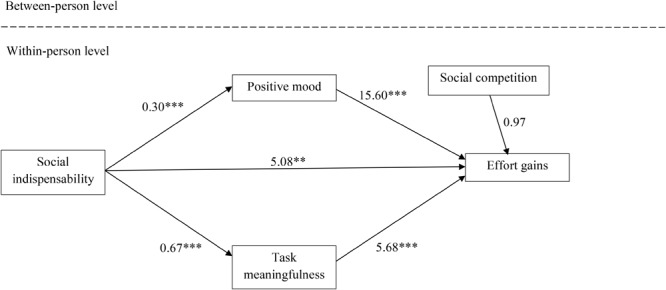
Mediation model (Study 1). Unstandardized coefficients are reported. ^∗^*p* < 0.05, ^∗∗^*p* < 0.01, ^∗∗∗^*p* < 0.001.

In order to examine the assumed mediation processes of social indispensability effects on effort gains in teams, we simultaneously considered mood (Hypothesis 3b) and perceived task meaningfulness (Hypothesis 4b) in our analysis. First, we compared a full-mediation model with a partial-mediation model. Because the partial-mediation model showed a better fit to the data [scaled Δ-2 × log-likelihood (1) = 9.57, *p* < 0.01], we retained this partial-mediation model for testing Hypotheses 3b and 4b (**Figure 1**). Further supporting the notion of partial mediation, the relationship between social indispensability and effort gains in teams (*b* = 5.08, *p* < 0.01) remained significant when the two mediators were entered into the model. Mood was contingent on the relationship between social indispensability and effort gains as indicated by a significant indirect effect quantified by the product-of-coefficient method (MacKinnon et al., 2002; coefficient = 4.67, z = 3.95, *p* < 0.01). These results supported Hypothesis 3b. Moreover, perceived task meaningfulness was contingent on the relationship between social indispensability and effort gains in teams (coefficient = 3.82, z = 3.90, *p* < 0.01), supporting also Hypothesis 4b.

Finally, we tested Hypotheses 3c and 4c, assuming that the relationship between perceived indispensability and positive mood (perceived task meaningfulness) is stronger than the relationship between social competition and positive mood (perceived task meaningfulness) in occupational teams. We regressed mood (perceived task meaningfulness) simultaneously on perceived indispensability and on social competition. Significant differences of the magnitudes of these relationships are indicated if the 90% confidence intervals (one-tailed testing) do not overlap. At the event level, perceived indispensability was a stronger predictor of positive mood (β = 0.29, *p* < 0.001, 90% CI: 0.18,0.40) than social competition (β = 0.06, *p* = 0.27, 90% CI: -0.03,0.14), supporting Hypothesis 3c. Similarly, perceived indispensability was a stronger predictor of perceived task meaningfulness (β = 0.44, *p* < 0.001, 90% CI: 0.36,0.53) as compared to social competition (β = 0.12, *p* < 0.01, 90% CI: 0.05,0.21), supporting Hypothesis 4c.

#### Effects on Strain

Finally, we explored how event-based social competition and social indispensability predicted perceived strain in the team events. Comparing the predictor effects between the two sources of team effort gains and strain in the same model revealed neither a significant result for event-specific social indispensability (β = 0.10, *p* = 0.16) nor for event-specific social competition (β = 0.07, *p* = 0.17). Similarly, the relationship between strain and effort gains was not significant (β = 0.09, *p* = 0.20).

### Discussion

Study 1 examined social competition and social indispensability as potential sources of effort gains in occupational teams using a within-subjects design. The results clearly supported the assumed effects of social indispensability, revealing event-specific perceptions of indispensability as significant predictor of effort gains in teams as compared to working alone. Please note that these event-specific effort gains would have been overlooked in a between-subjects design with effort ratings being aggregated across different job events. Moreover, the results showed that the motivating effects of perceived indispensability were correlated with affective (mood) and cognitive processes (perceived task meaningfulness). Although further research is needed, the current results provide the first evidence of mediating mechanisms of effort gains in existing occupational teams.

In contrast, no evidence was observed for event-specific social competition as a source of effort gains in occupational teams. This result suggests that motivating effects of social competition observed in laboratory teamwork (e.g., Seta, 1982; Stroebe et al., 1996) might not easily generalize to enduring occupational teams working for meaningful outcomes. However, the results are in line with our assumption that social competition is a less effective source of effort gains in occupational teams than social indispensability. Finally, the results provided no clear indication that event-based social indispensability or social competition perceptions were significantly correlated with experienced strain.

One limitation of Study 1 is the unexpected order effect, showing overall effort gains in teams (across specific events) only when participants started reconstructing working alone events as compared to participants starting with teamwork events. Although this order effect did not interfere with the other effects observed, it is worth to explore potential explanations. One explanation might be that the block-wise presentation of the different events to be reconstructed (three events working alone followed by three team events, or vice versa) might have implicitly suggested to contrast the two types of job events. Following conversation logic (e.g., Grice, 1975), this contrast might have caused an artificial increase of ratings in the second block. If this explanation is valid, the order effect might be avoided by eliminating a block-wise structure, and by stating explicitly that the order of the reconstructed events is assigned randomly. This was realized in Study 2.

Another limitation of Study 1 is that most reconstructed teamwork events referred to teams with relatively long team tenure (*M* = 7.5 years, *SD* = 8.1 years). Based on the assumption that social competition might be particularly motivating in earlier stages of team processes (Hypothesis 6), a replication study might be desirable that includes more teams in earlier phases of teamwork. Finally, participants of Study 1 were collected via an online panel. While the high diversity in occupational background provides a conservative test of our hypotheses, it might be desirable to replicate the observed findings in a more homogenous context.

## Study 2

The purpose of Study 2 was to replicate the main findings of Study 1 in a sample with a more homogenous occupational background. Moreover, the order variation of job events to be reconstructed was changed to avoid the observed order effect of Study 1. At the same time, we sought to include more teams with short team tenure to provide a more balanced test of Hypothesis 6. Finally, we assessed individual preferences for social competition and for teamwork to allow examinations of Hypotheses 7 and 8. All other features were similar to Study 1.

### Method

#### Participants

The study was conducted in eight small software developing companies. Out of 259 employees who started the questionnaire, 87 participants completed the survey resulting in a response rate of 33.6%, which can be considered above average in research using a within-subject design (e.g., Rothbard and Wilk, 2011). We excluded participants from analyses who reported working very rarely (5% or below of their time; *N* = 5) or nearly always in teams (95% or more of their time; *N* = 3), who reported the same ratings for all events (*N* = 4) or indicated the same date for all events (*N* = 5).^3^ The final sample consisted of 70 employees (13 women and 57 men; average age of 31.86 years, *SD* = 9.70, age range 18–69 years of age). Employees reported an average tenure of 1.9 years for the teams in the reconstructed job events (*SD* = 3.3) and spent about 48.8% (*SD* = 26.35) of their working time in teams. From this sample, 62.9% participants worked as software developers, 15.7% in product management, 7.1% in marketing, 7.1% in administration, 5.7% in sales, and 1.4% in management.

#### Procedure

Data was collected using the same online questionnaire as in Study 1. Similar to Study 1, we employed two different order conditions, with half of the participants starting with a teamwork event and the other half starting with a working alone event. However, to reduce order effects obtained in Study 1, the type of job event reconstructed – teamwork or working alone – was alternated in Study 2 after each job event. Moreover, we explicitly noted that the order of job events to be reconstructed was determined randomly by the online survey system.

#### Measures

We employed the same measures as in Study 1. Additionally, participants indicated their preference for social competition with three items (“I find competitive events unpleasant,” “I don’t like competing against other people,” and “I try to avoid competing with others”; α = 0.91) from the Competitiveness Index (Smither and Houston, 1992). Furthermore, participants reported their preference for teamwork with two items (“I prefer group work to individual work” and “Whenever possible, I like to work with others rather than by myself”; *r* = 0.63, *p* < 0.001) adopted from Karau and Elsaid (2009). Overall, Study 2 contained 47 items. Moreover, 60 additional items were collected in this survey that were part of a different research question.

#### Data Analyses

Data were hierarchically structured with work events (Level 1, *N* = [3 teamwork events × 70] = 210 events) nested within employees (Level 2, *N* = 70 employees). As in Study 1, we used multilevel modeling and conducted the analyses using Mplus 7.3 (Muthén and Muthén, 1998-2012) with robust maximum-likelihood estimation.

### Results

The changes made in Study 2 to reduce the observed order effect in Study 1 were successful, with a non-significant correlation between questionnaire order and effort gains in teams (*r* = 0.01, ns; see **Table 2**), and similar means of effort gains in teams in both employed conditions (*M*_Individual work first_ = 1.41, *SD* = 19.15; *M*_teamwork first_ = 2.10, *SD* = 28.48), *t* < 1. Therefore, we excluded questionnaire order from further analyses in Study 2. The ICC values indicated a substantial amount of variance at the within-person level for effort gains (60.3%), social competition (18.8%), social indispensability (65.7%), mood (61.4%), perceived task meaningfulness (56.2%), and strain (48.9%), supporting the use of multilevel analysis.

**Table 2:** Means, standard deviations, and correlations between study variables (Study 2; *N* = 70 Employees).

	Mean	*SD*	1	2	3	4	5	6	7	8	9	10	11	12	13	14	15
**Person-level variables**
(1) Order	0.56	0.50	–
(2) Gender	0.19	0.39	–0.02	–
(3) Age	31.86	9.70	–0.28*	0.09	–
(4) Working time in teamwork^a^	52.34	26.72	0.02	0.09	–0.29*	–
(5) Team tenure^b^	23.16	39.73	–0.19	–0.04	0.34**	–0.15	–
(6) Preference for social competition	3.80	1.72	0.20^†^	0.13	–0.17	0.00	–0.25*	–
(7) Preference for teamwork	4.99	1.13	0.06	0.04	–0.06	0.35**	–0.22^†^	0.23^†^	–
**Event-level variables**
(8) Social competition	4.50	1.87	0.13	0.16	–0.09	0.00	0.03	–0.01	–0.00	–	0.30***	0.01	0.12^†^	0.15*	0.08	0.10	0.01
(9) Social indispensability	5.44	1.01	–0.35**	0.07	0.14	–0.17	0.11	0.00	0.10	0.31**	–	0.05	0.31***	0.11	0.29***	0.04	0.17*
(10) Mood	4.93	0.92	–0.02	0.13	0.10	0.09	0.06	–0.17	0.19	–0.05	–0.10	–	0.37***	–0.35***	0.23**	0.61***	0.27***
(11) Meaningfulness	5.01	1.10	–0.07	0.14	0.09	–0.02	0.13	0.11	0.33**	0.08	0.31**	0.45***	–	0.14*	0.27***	0.47***	0.29***
(12) Strain	3.67	1.51	0.08	–0.29*	–0.14	0.06	0.04	0.13	–0.01	0.13	0.10	–0.38**	0.05	–	–0.27***	–0.18**	0.06
(13) Self-efficacy	5.68	0.91	–0.13	0.01	0.01	–0.08	0.18	–0.32**	–0.04	0.10	0.41***	0.23^†^	0.31**	–0.38**	–	0.28***	0.19**
(14) Motivation in teamwork	132.38	38.80	0.01	0.02	0.11	0.05	0.09	–0.01	0.34**	0.10	–0.05	0.67***	0.52***	–0.24*	0.25*	–	0.38***
(15) Effort gains	1.80	24.62	0.01	–0.14	–0.07	0.14	0.35**	0.07	0.35**	–0.03	0.11	0.12	0.23^†^	0.11	0.07	0.12	–

*Correlations below the diagonal are correlations at the person-level (N = 70) with aggregated event-level variables. Correlations above the diagonal are correlations at the event-level (N = 210). Order (0 = start with individual work, 1 = start with teamwork), gender (0 = male, 1 = female). ^a^Measured in percentage. ^b^Measured in months. ^†^p < 0.10, ^∗^p < 0.05, ^∗∗^p < 0.01, ^∗∗∗^p < 0.001*.

#### Social Competition, Social Indispensability, and Effort Gains in Teams

Similar to Study 1, the proposed effects of event-based social competition and social indispensability were tested in two steps. In the first step, we regressed event-specific effort gains in teams simultaneously on event-specific social competition and social indispensability ratings (see **Table 2**). In this analysis, only social indispensability significantly predicted effort gains in teams (*b* = 5.13, *SE* = 1.88, *p* < 0.01) whereas social competition effects failed to reach significance (*b* = 1.41, *SE* = 2.18, *ns*), replicating findings from Study 1 and again supporting Hypothesis 2 but not Hypothesis 1. Moreover, when constraining the relationships of social competition and social indispensability with effort gains in teams to be equal, and comparing this constrained model with the unconstrained model, the constrained and unconstrained models fit the data equally well [scaled Δ-2 × log-likelihood (1) = 1.64, *ns*]. Therefore, we favored the more parsimonious unconstrained model suggesting that the relationship between social indispensability and effort gains in teams is overall stronger than the relationship between social competition and effort gains in teams, which is in line with Hypothesis 5.

In a second step, we again examined whether high degrees of event-based social competition and/or social indispensability resulted in positive effort gains in teams, i.e., higher effort in team events as compared to working alone. Similar to Study 1, the overall mean of this difference score was close to zero (*M* = 1.80) with considerable variance (*SD* = 24.62; see **Table 2**), indicating the presence of both effort gains and losses in teams. We therefore again analyzed effort gains in teams separately for events with high and with low perceived social competition, and for events with high and with low perceived indispensability, both separations being based on median-splits. Effort gains in team events with high perceived indispensability were positive (*M* = 6.95, *SE* = 3.41) and significantly higher than zero, *t*(56) = 2.04, *p* < 0.05, with zero indicating equal effort ratings in teams and in working alone events. Effort gains in team events with low perceived indispensability were again negative (*M* = -5.21, *SE* = 4.26) although this time not significantly different from zero, *t*(44) = -1.22, *p* = 0.22. No significant effort gains or losses were observed for team events as a function of social competition being high or low. Thus, in addition to the positive interrelation between social indispensability and effort gains in teams, positive effort gains in teams as compared to working alone occurred again only when social indispensability was high in the occupational teams.

#### Testing Mediation Processes

**FIGURE 2:**
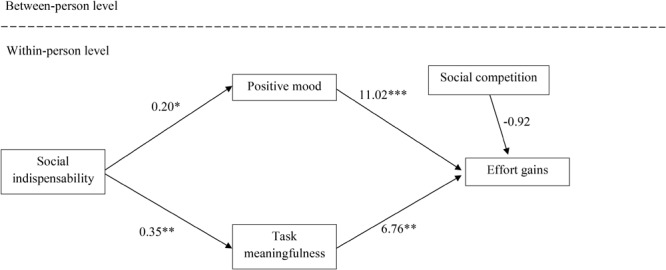
Mediation model (Study 2). Unstandardized coefficients are reported. ^∗^*p* < 0.05, ^∗∗^*p* < 0.01, ^∗∗∗^*p* < 0.001.

As in Study 1, we examined contingencies between event-specific effort gains in teams and the postulated mediation variables as initial indicators of mediation processes. Because event-based social competition ratings were unrelated to effort gains in teams, we again refrained from examining the respective mediation Hypotheses 3a and 4a, and included social competition ratings as control variable in the examinations of mediation processes between social indispensability and effort gains in teams (Hypotheses 3b and 4b). Similar to Study 1, we simultaneously tested mood and task meaningfulness as mediators of the relationship between social indispensability and effort gains. Because the full- and the partial-mediation models fitted the data equally well (scaled Δ-2 × log-likelihood (1) = 1.83, *ns*), we retained the more parsimonious full-mediation model for testing Hypotheses 3b and 4b in Study 2 (**Figure 2**). More specifically, the results revealed that positive mood was contingent on the relationship between social indispensability and effort gains in teams (coefficient = 2.24, *z* = 2.25, *p* < 0.05), suggesting mood as mediating process as predicted in Hypothesis 3b. Moreover, perceived task meaningfulness was also contingent on the relationship between social indispensability and effort gains in teams (coefficient = 2.34, *z* = 2.10, *p* < 0.05), in line with Hypothesis 4b. Finally, we tested Hypotheses 3c and 4c by again comparing the confidence intervals of the standardized relationships (one-sided tests). At the event level, perceived indispensability was significantly correlated with mood (β = 0.17, *p* < 0.05, 90% CI: 0.03,0.32) whereas social competition was not (β = 0.11, *p* = 0.14, 90% CI: -0.02,0.30). However, the overlapping confidence intervals provide no evidence that the magnitudes of the relationships differ significantly, this time providing no clear support for Hypothesis 3c. Similarly, the relationships of perceived indispensability (β = 0.21, *p* < 0.05, 90% CI: 0.06,0.36) and social competition (β = 0.15, *p* < 0.05, 90% CI: 0.04,0.26) with perceived task meaningfulness did not differ significantly, providing no support for Hypothesis 4c in Study 2.

#### Testing Moderation Processes

Different to Study 1, gender effects could not be explored in Study 2 because the sample consisted of mostly men. To examine the assumed context- and person-related moderators, we centered within-person predictor variables around each person’s mean value, and person-level moderator variables around the grand mean (Hofmann and Gavin, 1998). Consistent with Hypothesis 6, team tenure significantly moderated the relationship between social competition and effort gains as indicated by the cross-level interaction term (*b* = -1.36, *z* = -2.13, *p* < 0.05). To illustrate this moderation, we estimated the relationship between social competition and effort gains at the lowest value of team tenure (0 years; *b* = 3.55, *z* = 1.46, *p* = 0.07, one-tailed) and high values of team tenure (two standard deviations above zero: 6.6 years; *b* = -5.37, *z* = -1.42, *p* = 0.08, one-tailed), and found a significant difference between these two slopes (*b* = 8.91, *z* = 2.13, *p* = 0.03). The significant difference between the slopes indicates that social competition was a positive predictor of effort gains in teams in the beginning of teamwork, but this relation turned to be negative when team tenure was long. No such moderation was found for the relation between social indispensability and effort gains in teams.

**FIGURE 3:**
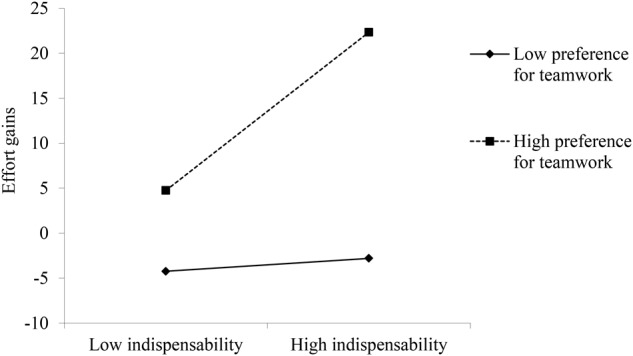
Study 2 cross-level moderation of preference for teamwork on the event-level relationship between social indispensability and effort gains in teams.

Again, no moderation effects of event-specific self-efficacy was found, neither linear nor curvilinear (*b*’s = 4.74 and -2.06 for the linear moderation, and *b*’s = -2.73 and -0.76 for the curvilinear moderation). **Table 3** illustrates the analyses for the other two person-related moderators. Hypothesis 7 postulated that the positive relationship between social competition and effort gains in teams is stronger for individuals with a high (vs. low) preference for social competition. However, this hypothesis was not supported because the cross-level interaction between event-level social competition and individuals’ preference for social competition did not predict effort gains in teams (*b* = 0.64, *ns*; Model 2 in **Table 3**). Hypothesis 8 proposed that the positive relationship between social indispensability and effort gains in teams is stronger for individuals with a high (vs. low) preference for teamwork. This hypothesis was supported by a significant cross-level interaction between event-level social indispensability and individuals’ preference for teamwork on effort gains in teams (*b* = 4.06, *p* < 0.01; Model 4 in **Table 3**). The pseudo-*R*^2^ change was 0.02 (from 0.10 to 0.12) after the interaction term was added to the model. Thus, the interaction term accounted for additional 2% of the total variance in team effort gains. To facilitate the interpretation of this cross-level moderation, we plotted the simple slopes for 1 *SD* above and 1 *SD* below the mean of the moderator variable. As displayed in **Figure 3**, social indispensability positively predicted effort gains in teams for individuals with a high preference for teamwork (*b* = 9.95, *z* = 4.14, *p* < 0.001), but not for individuals with a low preference for teamwork (*b* = 0.89, z = 0.43, *ns*). This result is in line with Hypothesis 8.

**Table 3:** Multilevel estimates for moderation models predicting effort gains (Study 2; *N* = 70 Employees).

	Model 1 (Hypothesis 7)			Model 2 (Hypothesis 7)			Model 3 (Hypothesis 8)			Model 4 (Hypothesis 8)		
Variables	Coeff.	*SE*	*t*	Coeff.	*SE*	*t*	Coeff.	*SE*	*t*	Coeff.	*SE*	*t*
Social competition	3.76	2.52	1.49	3.61	2.68	1.35						
Social indispensability							5.54	1.99	2.79^∗∗^	5.38	1.80	2.99^∗∗^
**Moderator variables**
Preference for social competition	0.93	2.00	0.47	0.93	2.00	0.47			
Preference for teamwork							7.58	2.57	2.95^∗∗^	7.58	2.57	2.95^∗∗^
**Cross-level interactions**
Social competition × preference for social competition				0.64	1.97	0.33
Social indispensability × preference for teamwork										4.06	1.22	3.32^∗∗^
-2 × Log likelihood (*df*)	2013.83 (5)		2013.26 (7)		1998.89 (5)		1991.55 (7)	
Scaling correction factor	1.37		1.30		1.24		1.19	
Scaled Δ–2 × log likelihood (Δ*df*)			0.51 (2)				7.03 (2)^∗^	
Level 1 error variance (*SE*)	591.35 (87.41)		553.86 (105.14)		565.87 (84.79)		537.79 (108.73)	
Level 2 error variance (*SE*)	400.30 (136.70)		410.56 (132.61)		338.50 (106.38)		345.47 (108.17)	
*Pseudo R*^2^	0.01		0.04		0.10		0.12	

*Unstandardized estimates are reported. Predictor variables on the event-level (social indispensability and social competition) were centered to each person’s mean. Order (0 = start with individual work, 1 = start with teamwork). Model 2 is compared to Model 1, Model 4 is compared to Model 3. ^∗^*p* < 0.05, ^∗∗^*p* < 0.01, ^∗∗∗^*p* < 0.001.*

#### Effects on Strain

We again explored the event-based contingencies between social competition and strain and between social indispensability and strain. Comparing social competition and social indispensability effects in the same model revealed this time a significant link between event-specific social competition and strain (β = 0.17, *p* = 0.01), whereas the link between event specific social indispensability and strain was non-significant similar to Study 1 (β = 0.04, *p* = 0.68). As in Study 1, the relationship between strain and effort gains was not significant (β = -0.02, *p* = 0.75).

### Discussion

Study 2 replicated the main findings of Study 1 using a more homogeneous sample with participants from one industry only and applying a changed order of the event reconstruction method. The changed order of the event reconstruction method successfully avoided main effects of order on effort gains in teams, lending initial support to our explanation of the observed order effect in Study 1. Moreover, the data again revealed clear effects of event-specific indispensability perceptions on effort gains in teams, supporting Hypothesis 2. The observed indispensability effects were again contingent on affective (mood) and cognitive (perceived task meaningfulness) process variables, providing further evidence for the assumed mediation proposed in Hypotheses 3b and 4b.

In contrast, no overall effects occurred for event-specific social competition, failing to support Hypothesis 1. Moreover, perceived competition in the teams was again less strongly correlated with perceived task meaningfulness than perceived indispensability. Positive correlations between event-based competition and effort gains in teams only occurred when team tenure was short, which is consistent with Hypothesis 6. Together with Study 1, the results of Study 2 suggest that effort gains in teams based on social competition are not easily found in enduring occupational teams. Instead, they seem to be most likely in early forming phases of teamwork. The resulting difference between event-based social indispensability and social competition as sources of effort gains in teams is in line with Hypothesis 5.

In addition to replicating the results of Study 1, Study 2 also examined two person-oriented moderation hypotheses that further speak to the assumed dynamics of social indispensability and social competition processes. In line with Hypothesis 8, the results showed that event-specific indispensability effects were particular found when persons had a high preference for teamwork (Karau and Elsaid, 2009), and were thus more likely to endorse communal values and collectivistic orientations which make them more susceptible for social responsibility norms. In contrast, a preference for competition (Smither and Houston, 1992) did not moderate effects of perceived event-based competition.

Finally, the explorative data of Study 2 showed significant correlations between event-based competition and strain experience even though no overall team effort gains were observed. In contrast, event-based indispensability perceptions correlated significantly with perceived effort gains in teams (and with task meaningfulness) but was uncorrelated with event-based strain. These findings further speak to our general assumption that social indispensability effects might be more stable and thus more effective in enduring occupational teams than social competition.

## General Discussion

This research examined if, when, and why teamwork is more motivating for individuals than working alone in everyday work settings. In doing so, we examined social competition and social indispensability – the currently most established sources of effort gains in teams – as potential predictors of higher effort during teamwork as compared to individual work settings. While no overall effort differences in teams and in individual work settings occurred across participants, the applied within-subjects design did reveal significant effort gains in teams under certain conditions. Thus, the current research goes beyond cross-sectional studies that ask participants to aggregate their experiences across different work events and conditions in order to rate their averaged effort. Instead, the current studies enabled a more differentiated look at job events within the same employee, considering instead of neglecting varying levels of event-based social competition and social indispensability.

The results of both studies consistently revealed significant effort gains in occupational teamwork as compared to working alone when event-specific indispensability perceptions were high. These results extend laboratory research that has demonstrated motivating effects of social indispensability in short-term ad-hoc teams (e.g., Hertel et al., 2000; Weber and Hertel, 2007; Kerr and Hertel, 2011). Moreover, the current results extend analyses of sports data that suggest indispensability effects within highly preselected samples of trained athletes performing maximum performance tasks in sequential action teams (e.g., Hüffmeier and Hertel, 2011b; Hüffmeier et al., 2012, 2017). The current research documents significant indispensability effects in teams for regular employees performing typical but also meaningful tasks in everyday situations.

Further extending prior knowledge, the current study provides first evidence of affective (i.e., mood) and cognitive (i.e., perceived task meaningfulness) processes that seem to mediate indispensability effects on team effort gains. These results are consistent with our assumption that perceived indispensability increases positive mood and task meaningfulness due to anticipation of pride and acknowledgment by others as well as collectivistic concerns and generic ingroup norms, which in turn should increase individuals’ willingness to invest higher efforts for the team. Moreover, the observed motivating effects of perceived indispensability occurred particularly when participants had a high (as compared to a low) preference for teamwork, which is also in line with our process assumptions.

In contrast, evidence that social competition triggers additional effort in occupational teams above and beyond individual work was only observed in Study 2 when teams were relatively new, and probably still establishing internal roles and hierarchies. Moreover, these effects were weaker than effects of event-based social indispensability. Motivating competition effects in teams thus seem to be more fragile because not all team members experience such comparisons positively, and/or because positive effects of social competition fade out over time when the comparison partners remain the same (e.g., Lount et al., 2008). Consistent with this conclusion, Lount and Wilk (2014) found performance-based evidence for motivating effects of social competition only when social competition was strongly facilitated by explicit performance postings, and even sank below the level of individual work later on. Please note that this conclusion is not in conflict with research documenting motivating effects of social competition in individual work settings (e.g., sales representatives competing for monetary bonuses). Our results only show that teamwork did not trigger stronger competition effects than individual work.

Together, the results of this research confirm that perceived indispensability can be a significant (and often underestimated) motivator in occupational teams. Moreover, the findings suggest that building on social responsibility and concerns for others might be a more effective motivation strategy in teams than merely relying on individualistic concerns to successfully compete with others (see also De Dreu, 2006; Grant and Berg, 2011; Hu and Liden, 2015). Notably, the current results did not replicate gender differences that have been found in earlier laboratory research (Weber and Hertel, 2007), suggesting that men and women are equally receptive to social indispensability cues in occupational settings. Indeed, Study 2 has replicated event-based social indispensability effects within a sample of mostly men.

### Limitations and Future Research

The reported research should be considered with the following limitations in mind. First, the collected data relied on retrospection and self-reports, and are therefore potentially affected by respective method biases such as self-presentation concerns, memory problems (e.g., due to emotional contents or individual differences, e.g., Abele and Gendolla, 2007; Bisby et al., 2018) and limited insights into non-conscious processes (e.g., Podsakoff et al., 2012). However, our focus on interaction effects and within-person variation instead of main effects should have reduced general problems of common method biases. Moreover, self-presentation concerns probably had no major influence because participants were approached online and had no direct contact with the researchers. In addition, the applied event reconstruction method specifically stressed episodic memory traces which should have led to richer and more accurate recall of momentary experiences than standard questionnaires (Kahneman et al., 2004; Hertel and Stamov-Rossnagel, 2013). Indeed, the fact that our data could document both social indispensability and social competition effects suggests that the event reconstruction method can capture both motivational sources. The focus of this research made it necessary to assess participants’ momentary experiences in different situations; these are difficult to obtain from external observations or supervisor ratings. Therefore, despite potential biases, self-reports provided important information to test our hypotheses (e.g., on mediation). Nevertheless, although moderate correlations between self-ratings and behavioral measures of effort gains in teams have been already observed in laboratory settings (e.g., Hertel et al., 2000, 2003a), it remains to be shown how strongly self-reported effort gains correlate with behavioral indicators or judgments by others in field settings. Moreover, not all involved processes might be consciously available, so that future research in field settings should also include non-responsive measures such as physiological and performance data.

As a second more general limitation, the cross-sectional nature of our data restricts the analyses of the assumed mediation processes. For instance, the current study cannot specify whether positive experiences correlated with social indispensability were a precursor (e.g., anticipated pride and personal relevance) or a consequence of event-specific effort gains in teams (e.g., experienced pride and personal relevance; see also Kerr and Hertel, 2011). Longitudinal analyses are required to disentangle these different possibilities. In a similar way, in Study 2 motivating effects of event-based indispensability in teams have been particularly found for employees with a high preference for teamwork. However, it remains to be examined whether these preferences were driven by stable personality dispositions or rather by context-dependent attitudes that are susceptible to personnel development interventions.

Third, we used single-item measures as part of the ERM procedure which raises the concern of low reliability. However, as Sackett and Larson (1990) pointed out, single-item measures may suffice if the measured constructs are rather narrow or unambiguous to the respondent. In contrast, multiple-item measures are typically recommended for more complex psychological constructs such as personality traits (Wanous et al., 1997). Indeed, researchers from various fields have found single-item measures useful for assessing constructs such as job satisfaction (Nagy, 2002; Dolbier et al., 2005), stress symptoms (Elo et al., 2003), mood (Kunin, 1955), and even self-esteem (Robins et al., 2001). For instance, Dolbier et al. (2005) found a very high correlation (*r* = 0.82) between a single-item and a 15-item measure of job satisfaction, and their results revealed almost identical relationships of the two job satisfaction measures with a set of theoretically related constructs. Given that the constructs considered in the ERM procedure were rather narrow and unambiguous, we felt that single-item measures are justifiable and advantageous to reduce the burden of our respondents. Indeed, Robins et al. (2001) argued that single-item measures are especially helpful in within-person studies where time constraints limit the number of items that can be administered. Nevertheless, we encourage future studies to use multiple-item measures to replicate and extend our findings. Moreover, larger sample sizes would be beneficial to increase the generalizability of our findings.

The current study focused on two potential sources of effort gains in occupational teams (i.e., social competition and social indispensability). This extends existing research in work settings that so far has focused only on social competition processes (Lount and Wilk, 2014). However, future research might also include other potential sources of effort gains in teams, such as intergroup competition (e.g., Erev et al., 1993, see Wittchen et al., 2011, for a review), social compensation (e.g., Williams and Karau, 1991), social facilitation (e.g., Aiello and Douthitt, 2001), or social support in teams (e.g., Hüffmeier and Hertel, 2011a; Hüffmeier et al., 2014). Measuring different sources of effort gains in teams simultaneously might also reveal contingencies and interactions between these different sources.

Finally, additional person factors as well as context conditions should be considered as moderators of motivating aspects of teamwork. Interestingly, no moderation by event-specific self-efficacy occurred in the current studies, suggesting that the observed effort gains are not restricted to persons with high or medium task capabilities. However, event-specific perceptions of self-efficacy are only a rough proxy for relative task capabilities in teams. Future research might also consider the distribution of self-efficacy within the team in addition to individual levels of self-efficacy. As another example, continuity of performance feedback has been shown as moderating factor for both social competition and social indispensability effects in laboratory teams (Kerr et al., 2007; Hertel et al., 2008) and might have also strong effects in occupational teams (e.g., Lount and Wilk, 2014). In fact, both social competition and social indispensability processes should be less likely and less effective when performance feedback is lacking. Finally, characteristics of team tasks deserve further consideration as potential triggers as well as moderators of social indispensability and social competition effects. For instance, task interdependence and coordination requirements might amplify social indispensability effects (e.g., Hertel et al., 2004; Van der Vegt and Van de Vliert, 2005), whereas high uncertainty might rather support social comparison mechanisms (e.g., Festinger, 1954; Navarro et al., 2014).

### Practical Implications

Perhaps the most important practical implication of the current research is to recognize the potential of social indispensability and related other-oriented motives in occupational teamwork. In both studies, perceived indispensability for the team proved to be a significant source of additional effort as compared to working alone, whereas social competition did so only to a limited degree. This result is consistent with earlier work stressing potential negative effects of competition in teams (e.g., Stanne et al., 1999). Managers of teams are therefore well advised to rely on mutual responsibility rather than competition in teams. In order to enable such indispensability effects, managerial tasks include developing a cooperative climate and responsibility norms, staffing teams in a way that each member truly is needed (i.e., not *dispensable*), and providing frequent feedback that clarifies the individual importance of each member’ contribution for the team success. However, team managers (or the team as a whole) are also responsible that indispensability effects are not exploited. Although social indispensability seems to be, on average, correlated with more positive experiences (mood, perceived task meaningfulness, lack of strain) than social competition, both social competition and social indispensability might sometimes lead to high pressure and even health risks in the long run (e.g., burnout). Thus, excessive levels and disproportionate distributions of social competition and social indispensability in a team should be avoided.

## Conclusion

The current research compared social competition and social indispensability as potential sources of higher effort in occupational teams as compared to working alone. Whereas only few traces of additional effort were observed due to social competition in teams, the results of both studies with employees from various branches and organizations revealed significant effort gains in teams when participants perceived themselves to be indispensable for the team. These results stress the power of social responsibility and concerns for others as motivating sources in occupational teams.

## Ethics Statement

Both studies reported in the manuscript have been conducted in accordance with the recommendations of the ethic committee of the Department of Psychology and Sports Science at the University of Mnster. Both studies included non-clinical surveys using non-invasive measures (self-ratings). No treatments or false feedbacks were given, and no potential harmful evaluation methods were used. Typical procedures of online research were applied in our studies. Accordingly, we followed the ESOMAR guidelines (https://www.esomar.org/publications-store/codes-guidelines.php) and the rules of the German Society for Online research (http://www.dgof.de/standesregeln/). Both guidelines especially stress anonymity, privacy and voluntariness of participants. In accordance with these, participation was completely voluntary and participants could drop out at any time without any negative consequences. Informed consent was obtained from all participants. All data were stored only using an anonymous ID for each participant.

## Author Contributions

GH contributed the conception of the study and wrote the first draft of the manuscript. GH, JH, KW, and OM specified study design and measures. KW, JP, and JF organized the database. CN performed the statistical analysis. All authors contributed to manuscript revision, read and approved the submitted version.

## Conflict of Interest Statement

The authors declare that the research was conducted in the absence of any commercial or financial relationships that could be construed as a potential conflict of interest.

## Footnotes

1 While the terms “motivation losses” and “motivation gains” have been used in the past to describe changes of individuals’ effort as a consequence of working on a team (e.g., Karau and Williams, 1993; Weber and Hertel, 2007), we prefer the terms “effort losses” and “effort gains” in teams as more precise descriptions of effort changes (i.e., changes in the intensity and persistence of behavior) due to teamwork. Motivation, in contrast, is a broader construct that not only includes effort but also direction of goal directed behavior (e.g., Geen, 1995).

2 Most participants followed the instruction and reconstructed work events from the last few days. For instance, 70% of the reconstructed team events dated back 7 days or less, and 96% within 1 month. Running the main analyses only on work events with a time delay of 1 month yielded virtually the same pattern as the analyses based on the complete sample, therefore we decided to keep all reconstructed events in the sample for power issues (maximum delay for team events was 103 days and for individual events 369 days in Study 1).

3 Similar as in Study 1, most participants followed the instruction and reconstructed work events from the last few days. Fifty-four percent of the reconstructed team events dated back 7 days or less, and 86% within 1 month. Running the main analyses only on work events with a time delay of 1 month yielded virtually the same pattern as the analyses based on the complete sample, therefore we decided to keep all reconstructed events in the sample for power issues.
